# Editorial Correspondence

**Published:** 1883-09

**Authors:** 


### Editorial Correspondence.

Berlin, August 9, 1883.

*Editors Buffalo Medical and Surgical Journal:*

Some time since, I wrote to you, promising to send something for the Journal, and, while I have nothing extraordinary to offer, I may yet, perhaps, give a few things which will be of interest as showing what is being done in different parts of Europe in relation to medical matters.

While in Zurich I visited the National Exposition, and found a department devoted to sanitary and medical matters. This division comprised the exhibits of the University of Geneva, some of which were of considerable interest. Among the most interesting was the department of Embryology, in charge of Prof. Fol, with photographs, projected upon glass, of microscopic enlargements. The department of Histology and Physiology, in charge of Prof. Etarnod, included about a hundred apparatuses, among which were many intended for the application of electricity and chemical reagents to specimens while under the microscope. Next to these was the laboratory for Comparative Anatomy of Prof. Karl Vogt, with an apparatus for testing the effect of constant movement upon the growth of animals. There was also a considerable number of anatomical preparations, the exhibit of Prof. Laskowski, and likewise a laboratory of Pharmacology, by Prof. J. Brun.

In addition to these there were the usual plans of hospitals, army medical equipments, health and mortality statistics, etc. Apparently great attention is paid, in Switzerland, to the correction of physical deformities. I noticed, in particular, two very complicated apparatuses, one called a Thoracograph, and the other, a Reproduction Apparatus, intended for the purpose of obtaining the outline of the chest and of fitting a perforated sole leather support without the employment of casts of gypsum or other material.

As you doubtless have had many reports in the various journals of the Hygienic Exposition, I shall only notice those things which particularly attracted my attention during a day's sojourn in the various buildings.

The Exposition is devoted to all that pertains to Hygiene and "des Rettungswesens," or life saving. Under these two heads the most unlimited latitude is given to the character of the exhibits. It is oftentimes difficult to imagine what bearing certain exhibits have toward either Hygiene or life saving. Certainly these two terms have been taken in their very broadest interpretation, and nothing which could in any way have any application has been excluded. Necessarily, the exhibition is very comprehensive and apparently is equally complete. The official catalogue is an octavo of nearly three hundred pages. As attracting marked attention may be mentioned the complete Crematorium erected by Siemens of Dresden. Access is obtained in three stories, thus showing the entire machinery, from the upper story, from which the body is lowered to the furnace, to the lower section, intended for the fuel. I may here say, that while at Milan I visited the one at that place. I was informed that over four hundred bodies had been here reduced to ashes, and the large number of receptacles, arranged in rows like the boxes in a post-office, verifies the statement.

Listerism and other disinfecting dressings are represented in all conceivable ways; also, disinfecting washing machines, for clothing and other articles. Of course, there are the usual number and variety of ventilators and heating apparatuses. But in respect to steam heating, the Americans are undoubtedly still far ahead, as this method of heating is but just beginning to be introduced. The large number and variety of maps and statistics pertaining to health and mortality, in relation to soil, drainage, population and social condition, proves the great importance attached to these conditions in their bearing on public health. Another department to which much attention is apparently devoted is animal vaccination; there are several complete exhibits, showing the methods employed and the results obtained; in one or more the animals themselves are exhibited. In the mining and iron working industries, Knapp the well-known manufacturer of cannons, exhibits fire-proof asbestos clothing. In this department are also a great many varieties of respirators for use in mines and by workers in elevators, etc. The evil influences of the continued use of the treadle of sewing machines seems to have been recognized in the invention of a water motor intended for attachment to an ordinary water pipe faucet. Among the appliances for use at the sick bed, a heat regulator deserves special mention. The purpose is to modify and regulate the local heat of any part of the body, by means of layers of flexible metal tubing folded in various shapes so as to fit the various parts of the body, and through which warm or cold water is allowed to pass. By means of caps for the head extremities, abdomen, etc., the part can be kept at any temperature desired, the whole being connected with a thermometer.

While at Vienna, I visited the anatomical and pathological museums, but these hardly admit of a description, and must be seen to be appreciated. I have also visited the Pathological Institute, in charge of Virchow, with its nearly one hundred thousand specimens. Here, also, are the collection of bones discovered at Troy, by Schiemann, and which were examined and brought here by Virchow. From here, we go to Halle, Leipsic, Heidelburg and again to Paris.

P. W. V. P.

